# Chronic kidney disease induces a distinct lipidomic signature and accelerates atherosclerosis progression in a novel minipig model

**DOI:** 10.1186/s42826-026-00274-w

**Published:** 2026-04-02

**Authors:** Marcelino Bermúdez-López, Paula Nogales, Manuel Martí-Antonio, Eva Castro-Boqué, Virtudes M. de Lamo, Laia Beà-Menchón, Sergio Luis-Lima, Esteban Porrini, Xavier Sanchez-Salguero, Mariona Jové, Elia Obis, Natàlia Mota-Martorell, Aurora Pérez-Gómez, Alicia Garcia-Carrasco, Milica Bozic, Jesús Guajardo, Carlos J. Pérez-Sánchez, Serafí Cambray, Núria Amigó, Reinald Pamplona, Jacob F. Bentzon, José M. Valdivielso

**Affiliations:** 1 Department of Experimental Medicine, University of Lleida (UdL), Lleida, Spain; 2 Vascular and Renal Translational Research Group, Institute for Research in Biomedicine of Lleida (IRBLleida), RICORS2040, Madrid, Lleida, Spain; 3 Centro Nacional de Investigaciones Cardiovasculares Carlos III (CNIC), Madrid, Spain; 4 Department of Clinical Medicine, Aarhus University, Aarhus, Denmark; 5 Department of Laboratory Medicine, Complejo Hospitalario Universitario de Canarias, Santa Cruz de Tenerife, Spain; 6 Instituto Tecnologías Biomédicas, University of La Laguna, Tenerife, Spain; 7 Department of Animal Science, ETSEAFIV, University of Lleida, Lleida, Spain; 8 Lleida Biomedical Research Institute (IRBLleida), Lleida, Spain; 9 Biostatistics Unit, Faculty of Veterinary Medicine, University of Extremadura, Cáceres, Spain; 10 Basic Medical Sciences Department, Serra Hunter Lecturer, University of Lleida (UdL), Lleida, Spain; 11 Biosfer Teslab, Reus, Spain. Department of Basic Medical Sciences, Universitat Rovira i Virgili (URV), Institut d’Investigació Sanitària Pere Virgili (IISPV), Reus, Spain; 12 Centro de Investigación Biomédica en Red de Diabetes y Enfermedades Metabólicas Asociadas (CIBERDEM), Instituto de Salud Carlos III (ISCIII), Madrid, Spain; 13 Centro Nacional de Investigaciones Cardiovasculares Carlos III (CNIC), Madrid, Spain; 14 Steno Diabetes Center Aarhus and Department of Cardiology, Aarhus University Hospital, Aarhus, Denmark

**Keywords:** Atherosclerosis, CKD, Contrast-enhanced ultrasound, Palmitic acid, Pig, Swine, Untargeted lipidomics, Vasa vasorum

## Abstract

**Background:**

Chronic kidney disease (CKD) markedly accelerates atherosclerosis, driving the excess cardiovascular morbidity and mortality in these patients. While rodent models have been indispensable for mechanistic studies, their small vessel size limits the use of non-invasive vascular imaging to monitor disease progression. Therefore, large-animal models are needed to bridge experimental insights with clinical applicability. We aimed to establish a novel minipig model of CKD to characterize the temporal changes of CKD-driven atherosclerosis. Six female Yucatan minipigs overexpressing a human gain-of-function PCSK9 mutant were randomized to CKD (*n* = 3) or control (*n* = 3) groups. CKD was induced by selective clamping of the left renal artery branches combined with contralateral nephrectomy using a minimally invasive laparoscopic approach. All animals were fed with a high-fat, high-cholesterol diet and followed for 15 months. Longitudinal assessments included vascular and renal ultrasound, computed tomography, plasma iohexol clearance for glomerular filtration rate (GFR) determination, and biochemical profiling of ions, cytokines, chemokines, and lipids, including advanced lipoprotein, lipidomic and fatty acid analyses. Between-groups differences were evaluated using effect sizes with 95% confidence intervals.

**Results:**

CKD pigs exhibited a significant reduction in GFR and increased blood creatinine. They exhibited accelerated atherogenesis, reflected by enhanced progressive adventitial vasa vasorum neovascularization in both carotid and iliac arteries, a higher burden of arterial calcifications in abdominal aorta and iliac arteries and postmortem larger atherosclerotic plaques and calcified areas in coronary arteries. CKD also altered the systemic inflammation profile (elevated IL-1ra, IL-2, IL-4, IL-8, and IL-10), promoted a proatherogenic lipoprotein phenotype with triglyceride-enriched VLDL, LDL, and IDL particles, increased VLDL particle number, and reduced LDL particle size. Lipidomic analyses revealed increased circulating and renal palmitic acid and distinct lesion-specific fatty acid signatures. Fatty streaks were enriched in palmitic acid and 10,16-dihydroxy-palmitic acid, and mature carotid plaques accumulated polyunsaturated fatty acids.

**Conclusions:**

This minimally invasive CKD model in gentically modified minipigs accelerates atherosclerosis and induces a unique lipidomic remodelling, providing a valuable translational platform to study kidney-vascular interactions and to test therapeutic interventions targeting CKD-driven atherosclerosis.

**Supplementary Information:**

The online version contains supplementary material available at 10.1186/s42826-026-00274-w.

## Background

Atherosclerosis, the accumulation of lipid-rich plaques within artery walls, is a leading cause of cardiovascular disease and is markedly accelerated in patients with chronic kidney disease (CKD) [1, 2]. CKD amplifies traditional cardiovascular risk factors and induces specific uremia-related metabolic and inflammatory changes that drive the exceptionally high cardiovascular morbidity and mortality in this population [3–6].

Translating findings from experimental models to the clinic remains challenging, particularly when relying on small rodents, which differ from humans in vascular histology, lipoprotein metabolism and metabolomic profiles [7]. Although, rodent models have been indispensable for elucidating atherogenic mechanisms [8], their diminutive vessels and atherosclerotic plaques preclude conventional non-invasive imaging studies of disease progression [9].

To overcome these limitations, transgenic Yucatan minipigs overexpressing the human gain-of-function mutant of proprotein convertase subtilisin/kexin type 9 (PCSK9^D374Y^) were generated [10]. These animals recapitulate familial hypercholesterolemia and develop accelerated, human-like atherosclerotic lesions [10]. The size and anatomy of porcine vessels permit vascular ultrasound, enabling serial, in vivo assessment of plaque initiation and progression without sacrificing study animals [11]. Vascular ultrasound is a non-invasive imaging technique routinely used in clinic that can detect atherosclerotic plaques, quantify burden, and improve cardiovascular risk stratification [12]. As in humans, pig arteries have an abundant adventitial vasa vasorum network [13], reinforcing their translational relevance. Indeed, increased vasa vasorum density is associated with plaque progression and vulnerability [14, 15]. Thus, the capacity to non-invasively monitor vasa vasorum dynamics in a living animal offers substantial translational relevance and aligns with a growing area of interest in human atherosclerosis research [16].

However, pig models incur substantial expenditures for specialized housing, veterinary oversight, surgical suites, and imaging facilities, rendering each study a considerable financial and ethical commitment [17]. To mitigate these challenges while maximizing scientific output, the implementation of the Three Rs principles are mandated in legislation and best-practice guidelines [18]. Currently, research is transitioning from sole reliance on binary *p*-value thresholds towards estimation-based inference. Reporting standardized effect sizes alongside confidence intervals provides a transparent measure of practical significance, informs power analysis, and helps avoid over- or under-powered studies [19, 20].

To date, four distinct surgical models of CKD have been stablished in pigs: the remnant kidney model via staged renal artery embolization followed by contralateral nephrectomy in juvenile pigs [21]; the bilateral renal artery stenosis model induced by endovascular coil placement [22]; the 5/6 nephrectomy model developed through two consecutive laparoscopic nephrectomies (partial followed by contralateral radical) in Bama pigs [23]; and the staged renal artery embolization model using autologous clots [24]. However, the role of CKD in driving atherogenesis and plaque progression remains uninvestigated in those swine models.

Thus, in the present study, we generated a novel CKD model in PCSK9-overexpressing minipigs by laparoscopic selective clamping of left renal artery branches, immediately followed by contralateral radical nephrectomy under the same anaesthetic session. We then characterized temporal changes in atherosclerosis over 15 months on a high-fat, high-cholesterol diet. Vascular and renal ultrasound were combined with adventitial vasa vasorum quantification, computed tomography, glomerular filtration rate measurement, and comprehensive blood profiling of electrolites, cytokines, chemokines, and lipid species (including conventional lipids, advanced lipoprotein subclasses, lipidomic signatures, and fatty-acid compositions). Group differences were evaluated using effect sizes with 95% confidence intervals to convey both magnitude and practical significance.

## Methods

### Animals

Animal procedures were approved by the Department of Territory, Housing and Ecological Transition of the Govern of Catalonia, Spain (Exp. Nos: 9768 and 9959). Six female Yucatan minipigs with hepatic overexpression of the human gain-of-function mutant of proprotein convertase/subtilisin kexin type 9 (PCSK9^D374Y^) [10] were housed at the Centre for Pig Studies (CEP, Lleida, Spain) for a two-month acclimation period. At 9 months of age, glomerular filtration rate was assessed, vascular and renal ultrasound examinations were performed, and a blood samples were collected for analyses of ions, cytokines, chemokines, and lipids (including conventional lipids, advanced lipoprotein profiles, lipidomic signatures, and fatty-acid compositions) to establish baseline values. Animals were then randomized to either the CKD intervention group (*n* = 3), or the control group (*n* = 3) and transferred to the pig facilities of the Institute of Biomedical Research of Lleida (CREBA, IRBLleida, Lleida, Spain).

To induce CKD, intervention-group animals underwent laparoscopic selective clamping of left renal artery branches, immediately followed by contralateral radical nephrectomy under the same anaesthetic session. One week post-operation, pigs returned to the Centre for Pig Studies for clinical follow-up. Three weeks after surgery, all animals were switched from a standard diet to a high-fat, high-cholesterol (HF-HC) diet. HF-HC diet was conventional feed supplemented with 20% (w/w) lard and 2% (w/w) cholesterol (Sigma-Aldrich) until termination. HF-HC diet was prepared by dissolving cholesterol in melted lard before mixing. Feeding was ad libitum until pigs reached 35–40 kg and thereafter restricted to 700 g per day in two equal portions. Animals were monitored daily by facility staff throughout the 15-month study period. All endpoint assessments were performed under blinded conditions. Blood sample collection, processing and biochemical analyses were carried out by technicians who were unaware of the experimental group assignments. Histological evaluations were performed by an investigator blinded to intervention allocation, and all imaging analysis (ultrasound and post-mortem imaging) were conducted by experienced analysts who did not have access to the group identities.

### Laparoscopic surgery

CKD pig underwent laparoscopic surgery under general anaesthesia. Animals were sedated with intramuscular tiletamine-zolazepam (4 mg/kg each; Zoletil 100; Virbac, Barcelona, Spain) and xylacine (2.2 mg/kg; Xilagesic 20%, Calier, Barcelona, Spain), using a 19-gauge needle with extension set to minimize stress. Once sedated, pigs were transferred to the pre-operative room for washing, then received intramuscular atropine (0.01 mg/Kg; Atropina B. Braun 1 mg/ml, B. Braun Medical, Barcelona, Spain) and had a transdermal fentanyl patch (75 mg/h; Durogesic Matrix, Janssen-Cilag, Madrid, Spain) applied. During these preparations, animals were pre-oxygenated with 100% O_2_ at 4 L/min via mask, then moved to the operation room.

To prevent hypothermia, pigs were placed over a warning blanket, and two peripheral venous catheters (marginal ear veins) plus one arterial line (ear artery) were secured. An endotracheal tube was inserted under sevoflurane anesthesia (Sevoflurane 100%, Baxter, Lessines, Belgium; 4% vol/vol in O_2_ via mask). After intubation, sevoflurane was reduced to 2.5% and mechanical ventilation was initiated. Immediately pre-incision, pigs received three IV boluses: Propofol (2.5 mg/kg; Propofol Fresenius 10 mg/ml, Fresenius Kabi, Bad Homburg, Germany), fentanyl (5 mg/kg; Fentanest 0.05 mg/ml, B. Braun, Barcelona, Spain), and rocuronium (0.2 mg/kg; Rocuronio B. Braun, Melsungen, Germany). Heart rate, blood pressure, respiratory rate, SpO_2_, tidal volume, end-tidal CO_2_, inspired/exhaled sevoflurane and core temperature were continuously monitored.

To induce CKD, left renal hilum was exposed and the two main first-order branches of the renal artery were identified: the cranial polar artery and the caudal polar artery. We then selectively clamped the cranial polar artery and the anterior segmental branch of the caudal polar artery. Intraoperative renal Doppler ultrasound confirmed marked reduction of renal perfusion. Immediately thereafter, in the same anaesthetic session, a contralateral radical nephrectomy was also performed. Laparoscopic instruments (Karl Storz, Tuttlingen, Germany), an electrosurgical unit (Covidien, Dublin, Ireland), and automatic suturing devices were used.

For intra- and postoperative analgesia, a continuous IV infusion (10 ml/kg/h) of morphine (24 mg/L; Morfina B. Braun 10 mg/ml, Jaén, Spain), lidocaine (300 mg/L; Lidocaína Clorhidrato 5% Fresenius Kabi), and ketamine (60 mg/L; Ketamidor 100 mg/ml, VetViva Richter, Wels, Austria) was administered. After surgery, pigs recovered in a dedicated reanimation room. In the immediate postoperative period (3 days) animals also received flunixin meglumine (2.2 mg/kg) in addition to the fentanyl patch.

### Glomerular filtration quantification

Animals remained conscious, unsedated and were free to move throughout. After an 8-hour fast, a single 10 mL dose of Omnipaque 350 (350 mg/ml iohexol, GE Healthcare Bio-Sciences Corp, Madrid, Spain) was injected via one marginal auricular vein [25]. Blood samples (10 mL each) were then collected from the contralateral marginal auricular vein using a heparinized micro-capillary pipette (Hirschmann^TM^, Fischer Scientific, Madrid, Spain) onto Whatman 903 filter paper (GE Healthcare Bio-Sciences Corp, Madrid, Spain) at 0 (pre-dose), 120-, 180-, 240-, 300-, 360,- 420-, and 480-minutes post injection. Plasma iohexol concentrations and glomerular filtration rate were determined as previously described [26].

### Renal ultrasound

Pigs were sedated with intramuscular midazolam (0.6 mg/kg; Midazolam 5 mg/ml, Normon, Madrid, Spain), ketamine (8 mg/Kg; Ketaset 100 mg/ml, Ecuphar, Barcelona, Spain), and xylacine (2.2 mg/Kg; Xilagesic 200 mg/mL, Calier, Barcelona, Spain). A 21-gauge needle with extension set facilitated injection. Renal ultrasound was performed using a VIVID *i* BT09 system (GE Healthcare, Madrid, Spain) with a 4C-RS convex probe (1.5–6 MHz) and pulsed-Doppler to assess hemodynamics. Cortical thickness was measured at the lateral border and at the upper and lower poles. The arterial resistive index was determined in the main renal artery and in the interlobar arteries of both the upper and lower pole.

### Contrast-enhanced ultrasound (CEUS)

Pigs were sedated with intramuscular midazolam (0.6 mg/kg; Midazolam 5 mg/ml, Normon, Madrid, Spain), ketamine (8 mg/Kg; Ketaset 100 mg/ml, Ecuphar, Barcelona, Spain), and xylacine (2.2 mg/Kg; Xilagesic 200 mg/ml, Calier, Barcelona, Spain). A 21-gauge needle with extension set facilitated injection. CEUS was performed to assess adventitial vasa vasorum using a Siemens Acuson Sequoia 512 system (Siemens Healthineers España, Madrid, Spain) equipped with a 15-MHz linear array probe at a low mechanical index (0.4) and cadence contrast pulse sequencing software. SonoVue (Bracco, Milan, Italy) was reconstituted in 5 mL of saline, and a 2.5-mL bolus, followed by a 10-mL saline flush, was injected via the marginal ear vein for each artery. This dose provided a clear signal for ~ 1 minute. Vasa vasorum signal intensity was measured on the far wall of the common carotid artery and iliac artery, 1 cm proximal to their bifurcations. CEUS data were analysed as previously described [27]. Briefly, vasa vasorum intensity was quantified as the ratio of signal intensity in the 2 mm above the intima-lumen boundary to that in the 2 mm region below the media-adventitia boundary, measured 1 cm proximal to the bifurcation on the far wall. Because the contrast intensity varies with both time and animal, ratios were calculated frame by frame within periods of stable intensity. For each artery, ten independent frames were selected from the one-minute DICOM examination video for quantitative analysis.

### Carotid artery ultrasound

It was performed in both common carotid arteries. The VIVID *i* BT09 system (GE Healthcare, Madrid, Spain) with a 12 L-RS linear probe (6–13 MHz) and a pulsed-Doppler ultrasound to assess hemodynamics was used.

### Computed tomography

Procedures were performed under general anesthesia. Pigs received intramuscular methadone (0.5 mg/kg; Semfortan 10 mg/ml, Esteve, Barcelona, Spain) and dexmedetomidine (0.5 mg/kg; Dexdomitor 0.5 mg/ml, Zoetis Spain, Madrid, Spain) as premedication. An IV bolus of propofol (1 mg/kg; Propovet 10 mg/ml, Ecuphar veterinaria, Barcelona, Spain,) induced anesthesia, which was maintained with isoflurane (Isoflo 100% p/p, Ecuphar, veterinaria, Barcelona, Spain) via endotracheal tube. Tomographic images were acquired on a third-generation, 16-slice Hitachi Supria CT Scanner using a standard medium-frequency logarithm protocol for both native and venous-phase scans. Contrast (Omnipaque 350, 350 mg/ml iohexol, GE Healthcare Bio-Sciences Corp, Madrid, Spain) was injected manually through 18-gauge catheters placed in the right and left marginal auricular veins. Pigs laid in sternal decubitus with the anterior and posterior limbs extended. Scan parameters: whole body: 120 kV, 250 mA, 2.5 mm slices; carotids: 120 kV, 250 mA, 0.63 mm slices; thorax: 140 kV, 300 mA, 0.63 mm slices; abdominal aorta: 120 kV, 250 mA, 1.25 mm slices; and Iliac arteries: 140 kV, 300 mA, and 0.63 mm slices pitch 0.815, rotation time 0.6 s. A soft tissue window (WW 350, WL 40) was used. 3D multiplanar (sagittal and dorsal) reconstructions and volume renderings were generated. DICOM datasets were anonymized and reviewed on Osirix MD (v0.9.0.1, Pixmeo SARL, Bernex, Switzerland) by a single blinded radiologist

### Histology

Pigs were sedated with intramuscular midazolam (0.6 mg/kg; Midazolam 5 mg/ml, Normon, Madrid, Spain), ketamine (8 mg/Kg; Ketaset 100 mg/ml, Ecuphar, Barcelona, Spain), and xylacine (2.2 mg/Kg; Xilagesic 200 mg/ml, Calier, Barcelona, Spain), then heparinized (10,000 IU; Heparin 5000 IU/ml, Laboratorios Reig Jofré, Barcelona, Spain) to prevent coagulation. Euthanasia was performed under deep anesthesia with pentobarbital (4 mL/kg; Eutanax 200 mg/mL, Fatro Ibérica, Barcelona, Spain). The heart, aorta and iliofemoral arteries were fixed in 4% phosphate-buffered formaldehyde for 6 hours, then stored in cold phosphate-buffered saline. Serial sections of the target arteries were excised for histological analysis. The proximal 3 cm of the right coronary artery, left anterior descending artery, and circumflex artery were divided into six 5-mm sections. The proximal 10 cm of the right common iliac artery and its extension into the external iliac artery and femoral artery were divided into 1 cm sections. Samples were paraffin-embedded, sectioned in 3-mm sections, and stained with Masson’s trichome and Von Kossa to measure atheroma plaque area and calcification area, respectively. Lesions areas were quantified using ImageJ (NIH) by a blinded observer [28].

### Blood collection

Pigs were sedated with intramuscular midazolam (0.6 mg/kg; Midazolam 5 mg/ml, Normon, Madrid, Spain), ketamine (8 mg/Kg; Ketaset 100 mg/ml, Ecuphar, Barcelona, Spain), and xylacine (2.2 mg/Kg; Xilagesic 200 mg/ml, Calier, Barcelona, Spain). A 21-gauge needle with extension set facilitated the injection. Blood samples were collected from marginal ear vein after cleaning the site with 70% ethanol and povidone-iodine. The vein was occluded at the base of the lateral ear, a 21-gauge needle was inserted towards the ear base, and blood was collected in serum and plasma BD vacutainer tubes (Becton Dickinson, Madrid, Spain).

### Conventional biochemical parameters

Serum samples were collected after a 6-hour fast. Commercial reagents for use in Beckman Coulter AU analysers (Beckman Coulter, Nyon, Switzerland) were employed to quantify: total cholesterol (cholesterol oxidase/esterase/peroxidase method); triglycerides (lipase/glycerol kinase/colorimetric method); LDL-Cholesterol (cholesterol oxidase/esterase/peroxidase method); sodium, potassium, and chloride (indirect potentiometry); creatinine (enzymatic, creatinine amidohydrolase method); calcium (arsenazo III method); phosphorus (phosphomolybdate method).

### Cytokine and chemokine profile

Serum concentrations were measured using the MILLIPLEX MAP Porcine Cytokine and chemokine magnetic bead panel immunology multiplex assay (PCYTMAG-23K, Merk Millipore, Darmstadt, Germany) in accordance with the manufacturer’s instructions.

### Advanced lipoprotein profile

1 H-NMR spectra were recorded at 310 K on a Bruker Avance III 600 spectrometer (Bruker BioSpin GmbH, Rheinstetten, Germany) operating at a proton frequency of 600.20 MHz (14.1 T) in Biosfer Teslab (Reus, Spain). Serum lipid concentrations (triglycerides and cholesterol), particle size and particle number of the four main lipoproteins classes (intermediate-density lipoprotein (IDL), very-low-density lipoprotein (VLDL), low-density lipoprotein (LDL), and high-density lipoprotein (HDL), as well as the particle number of nine subfractions (large, medium and small VLDL, LDL, and HDL) were determined as previously described [29]. Subfraction particle concentrations were calculated by dividing the lipid volume by the particle volume for each class; lipid volumes were obtained using established conversion factors to convert concentration units into volume units [30].

### Untargeted lipidomics by LC/MS

Lipidomics analysis was performed as previously described [31]. Sample preparation: fresh-frozen tissues were collected. For the lipid extraction, 10 μL of the homogenized tissue were mixed with 5 μL of MiliQ water and 20 μL of ice-cold methanol. Samples were vigorously shaken by vortexing for 2 min, and then 250 μL of methyl tert-butyl ether (MTBE), containing internal lipid standards were added. Samples were immersed in a water bath (ATU Ultrasonidos, Valencia, Spain) with an ultrasound frequency and power of 40 kHz and 100 W, respectively, at 10 °C for 30 min. Then, 25 μL MiliQ water were added to the mixture, and the organic phase was separated by centrifugation (1.400 ×g) at 10 °C for 10 min. Sample injection in a LC-MS system: lipid extracts were subjected to liquid chromatography-mass spectrometry using a UPLC 1290 series coupled to ESI Q-TOF MS/MS 6545 (Agilent Technologies, Barcelona, Spain). The sample compartment of the UHPLC was refrigerated at 4 °C, and for each sample, 10 μL of lipid extract was applied onto a 1.8 μm particle 100 × 2.1 mm id Waters Acquity HSS T3 column (Waters, Milford, MA, USA) heated at 55 °C. The flow rate was 400 μL/min with solvent A composed of 10 mM ammonium acetate in acetonitrile-water (40:60, v/v) and solvent B composed of 10 mM ammonium acetate in acetonitrile-isopropanol (10:90, v/v). The gradient started at 40% of mobile phase B, reached 100% B in 10 min, and held for 2 min. Finally, the system was switched back to 40% of mobile phase B and was equilibrated for 3 min. Duplicate runs of the samples were performed to collect positive and negative electrospray-ionized lipid species in a TOF mode, operated in full-scan mode at 100 to 3000 m/z in an extended dynamic range (2 GHz), using N2 as nebulizer gas (5 L/min, 350°C). The capillary voltage was set at 3500 V with a scan rate of one scan/s. Continuous infusion using a double spray with masses 121.050873, 922.009798 (positive ion mode) and 119.036320, 966.000725 (negative ion mode) was used for in-run calibration of the mass spectrometer. Lipidomic data pre-processing and annotation: MassHunter Qualitative Analysis Software (Agilent Technologies, Barcelona, Spain) was used to obtain the molecular features of the samples, representing different co-migrating ionic species of a given molecular entity using the Molecular Feature Extractor algorithm (Agilent Technologies, Barcelona, Spain). MassHunter Mass Profiler Professional Software (Agilent Technologies, Barcelona, Spain) and Metabolanalyst Software [32] were used to perform a non-targeted lipidomic analysis of the obtained data. Only those features with a minimum of 2 ions were selected. After that, the molecular characteristics in the samples were aligned using a retention time window of 0.1% ± 0.25 min and 30.0 ppm ±2.0 mDa. Only features found in at least 70% of the QC samples accounted for the correction of individual bias, and the signal was corrected using a LOESS approach. For annotation, relevant features, defined by exact mass and retention time, were searched against the HMDB [33] (accuracy < 30 ppm) and LIPID MAPS databases [34] (accuracy < 20ppm). The identities obtained were compared to the authentic standards’ retention times. Finally, identities were confirmed by searching experimental MS/MS spectra against in silico libraries, using HMDB [33] and LipidMatch [35], an R-based to tools for lipid identification.

### Fatty acid composition by GC: fatty acids extraction

Total lipids from homogenates samples were extracted into chloroform:methanol (2:1, v/v). The chloroform phase was evaporated under nitrogen, and the fatty acyl groups were transesterified by incubation in 2.5 mL of 5% (v/v) methanolic HCl at 75 °C for 90 min. The resulting fatty acid methyl esters were extracted by adding 1 mL of saturated NaCl solution and 2.5 mL of n-pentane. The n-pentane phase was separated and evaporated under N2. The residue was dissolved in 50 µL of CS2, and 2 µL were used for analysis. Injection in a GC system: Separation was performed by a DBWAX capillary column (30 m ×0.25 mm ×0.25 μm) in a GC System 7890A with a Series Injector 7683B and an FID detector (Agilent Technologies, Barcelona, Spain). The sample injection was in splitless mode. The injection port was maintained at 250 °C, and the detector at 250 °C. The program consisted of 1 min at 150 °C, followed by 25°C/min to 180 °C, 10°C/min to 200 °C, 5°C/min to 220 °C, and finally 10°C/mint to 230 °C for 5 min, with a post-run at 250 °C for 10 minutes. The total run time was 16.2 minutes. Identification of fatty acid methyl esters was made by comparison with authentic standards (Larodan Fine Chemicals, Malmö, Sweden) using specific software of data analysis for GC from Agilent (OpenLAB CDS Chem Station v. C0.01.10; Agilent Technologies, Barcelona, Spain) and subsequent expert’s revision and confirmation. Results are expressed as mol%. Indices calculation: the following fatty acyl indices were also calculated: saturated fatty acids (SFA); unsaturated fatty acids (UFA); monounsaturated fatty acids (MUFA); polyunsaturated fatty acids (PUFA) from *n*-3 and *n*-6 series (PUFAn-3 and PUFAn-6, respectively); dimethyl acetal (DMA); and average chain length, ACL = [(Σ%Total14 ×14) + (Σ% Total16 × 16) + (Σ%Total18 × 18) + (Σ%Total20 × 20) + (Σ% Total22 × 22) + (Σ% Total24 × 24)]/100. The density of double-bonds was calculated with the Double-Bond Index, DBI = [(1 × Σmol% monoenoic) + (2 × Σmol% dienoic) + (3 × Σmol% trienoic) + (4 × Σmol% tetraenoic) + (5 × Σmol% pentaenoic) + (6 × Σmol% hexaenoic)]. Lipidome susceptibility to peroxidation was calculated with the Peroxidation Index, PI = [(0.025 × Σmol% monoenoic) + (1 × Σmol% dienoic) + (2 × Σmol% trienoic) + (4 × Σmol% tetraenoic) + (6 × Σmol% pentaenoic) + (8 × Σmol% hexaenoic)].

### Statistical analysis

#### Sample size calculation

A priori sample size estimation indicated that, with three pigs per group, a one-sided significance level (α) of 0.10, and a hypothesized effect size of 2.0 (Glass’s Δ or Cohen’s d, classified as “huge” according to Sawilowsky’s criteria [36]), the statistical power achieved was 81.46%.

#### Descriptive analysis

For all quantitative variables, mean and standard deviation or 95% CI were computed for control (CTRL) and CKD intervention groups at multiple timepoints. Animal weight, glomerular filtration rate, and vasa vasorum density were normalized to baseline values by dividing each measurement by its corresponding value at 0 time.

#### Comparative analysis

Given the limited sample size (n_CTRL_ = n_CKD_ = 3), conventional hypothesis testing was deemed underpowered [37]. Instead, group comparisons at each timepoints were performed using effect sizes to estimate the magnitude of differences, which are less sensitive to sample size variations [38]. Two effect size estimators were applied:

**A) Glass’s delta (∆):** Used when comparing one group mean against a reference group mean [39–41]:

$$\Delta \; = \;{{{{\overline X }_{CKD}} - {{\overline X }_{CTRL}}} \over {{S_{CTRL}}}}\;,$$

Where X_CKD_ and X_CTRL_ are group means and S_CTRL_ is the standard deviation of the control group. 95% confidence intervals for D wecon [42, 43]. A difference was considered statistically significant when the CI did not include zero.

**B) Cohen’s d:** Applied when S_CTRL_ = 0, using pooled variance estimates for two independent samples [44]:

$$d\; = \;{{{{\overline X }_{CKD}} - {{\overline X }_{CTRL}}} \over {{S_p}}}\;,$$

S pooled:

$${S_p}\; = \sqrt {{{\left( {{n_{CTRL}} - 1} \right)S_{CTRL}^2 + \left( {{n_{CKD}} - 1} \right)S_{CKD}^2} \over {{n_{CTRL}} + {n_{CKD}} - 2}}} \;.$$

95% confidence intervals of d were calculated analogously to ∆.

#### Lipidomic analysis

A total of 752 lipid metabolites were quantified in serum and tissue samples. ∆ and d effect sizes with 99% confidence intervals were computed to account for multiple comparisons. Metabolites were considered differentially abundant if significant differences were observed in serum and in at least one atheromatous lesion (fatty streak or atheroma plaque in the aortic arch, iliac artery or carotid artery) with changes in the same direction (X_CTRL_ ≤ X_CKD_ or X_CTRL_ ≥ X_CKD_). These metabolites were further examined in liver, adipose tissue, muscle, urine and kidney using the same criteria.

#### Fatty acids analysis

Twenty-seven fatty acids and eleven lipid indexes were analysed in serum and tissue samples. ∆ and d effect sizes with 99% confidence intervals were computed, including subtotal indexes. For atheromatous lesions, only fatty acids showing consistent directional differences with serum were reported (CTRL ≤ CKD or CTRL ≥ CKD). Additional analyses were performed on liver, adipose tissue, muscle, urine and kidney.

#### Software

All analyses were performed using R software [45]. Plots were generated with the ggplot2 library [46], accounting for the non-central t-distribution, and Microsoft Excel 365 was used for tabular visualization.

## Results

### A novel laparoscopic porcine CKD model

**Fig. 1:**
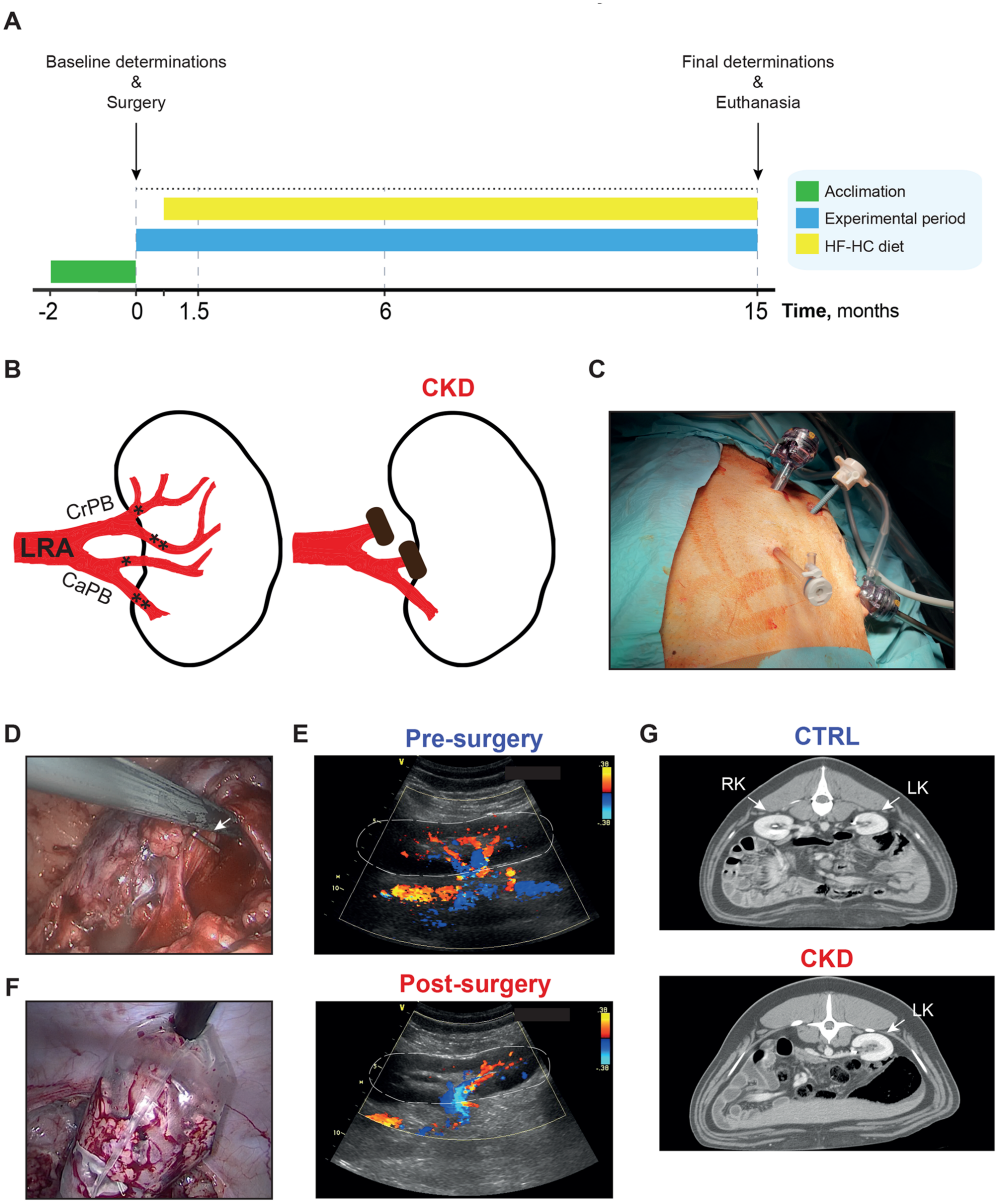
Porcine CKD study: (**A**): experimental timeline and animal interventions. (**B**): schematic of the left kidney showing selective clamping of the cranial polar branch (crPB) and the anterior segmental branch of the caudal polar branch (CaPB) of the left renal artery (LRA). (**C**): laparoscopic port configuration for instrument triangulation. (**D**): intraoperative view of the CrPB clamping. (**E**): intraoperative Doppler ultrasound demonstrating marked reduction in perfusion of the upper Pole and mid-region following vascular clamping. (**F**): intra-operative image of the contralateral radical nephrectomy. (**G**): contrast-enhanced CT scan confirming the absence of the right kidney and presence of vascular staples at the left renal artery site. *: anterior segmental branch. **: posterior segmental branch

Six female PCSK9^D374Y^ minipigs were randomized to CKD intervention (*n* = 3) or control (*n* = 3) groups. The surgical protocol (Fig. 1A) included laparoscopic selective clamping of the left renal cranial polar artery and the anterior segmental branch of the caudal polar artery (Fig. 1B–D). Intraoperative ultrasonography confirmed a marked reduction in renal perfusion (Fig. 1E), after which a contralateral radical nephrectomy was performed within the same anesthetic session (Fig. 1F). Postoperative contrast-enhanced CT scan verified right kidney removal and vascular staples at the left renal artery site in CKD animals (Fig. 1G).

**Fig. 2:**
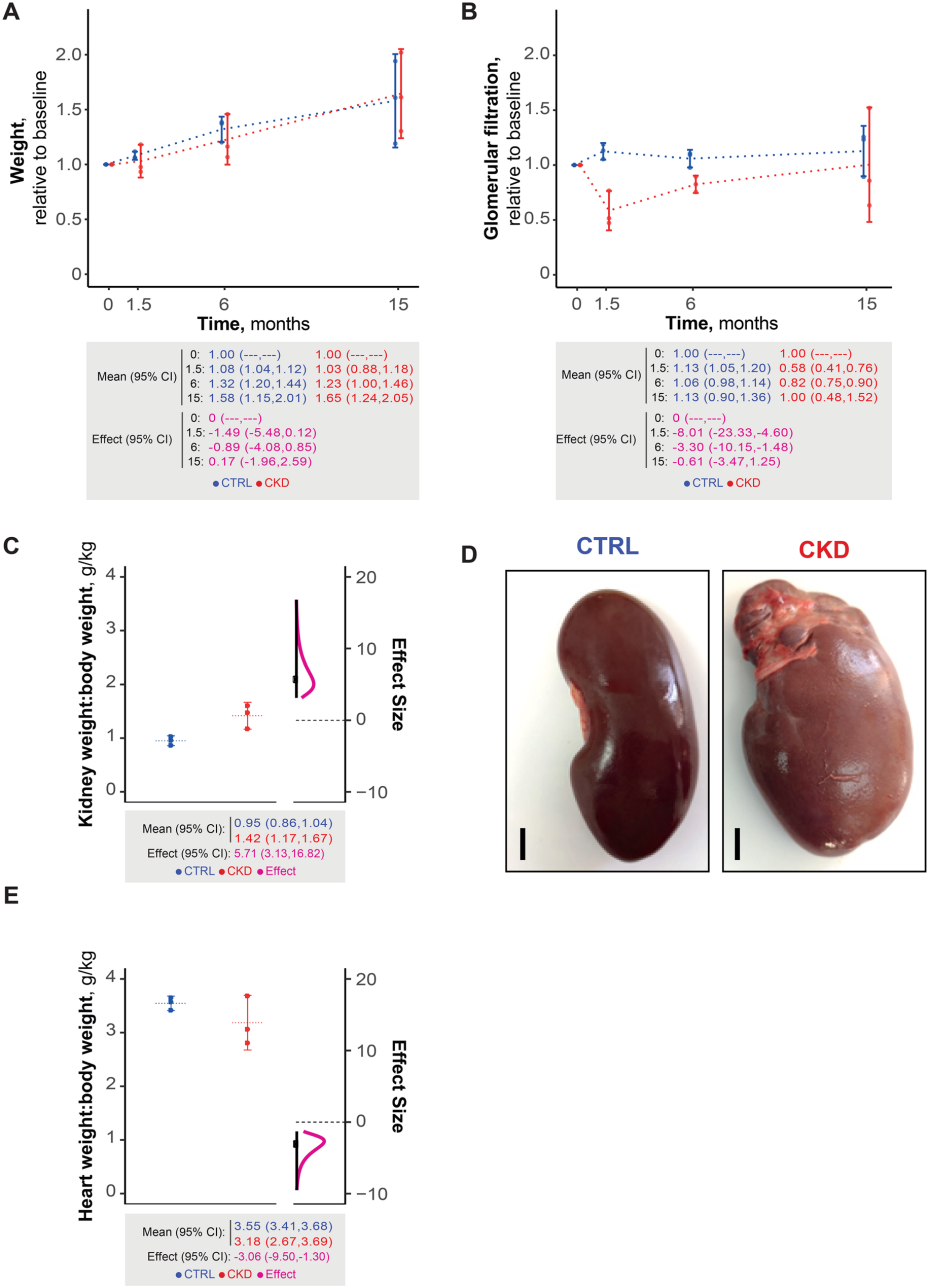
Porcine CKD model characterization: (**A**): longitudinal body-weight changes, expressed relative to baseline weight, over 15 months. Top: individual values, group means with 95% CI. Bottom: summary mean and effect size with 95% CI. (**B**): glomerular filtration rate normalized to baseline, over 15 months, plotted as in A. (**C**): post-mortem left kidney/body weight ratio. Left: individual values, group means with 95% CI. Right: distribution of effect size with 95% CI. Bottom: summary mean and effect size with 95% CI. (**D**): Representative post-mortem images of left kidneys, showing ccompensatory hypertrophy and cortical scarring at the upper Pole. Scale bar = 1 cm. (**E**): post-mortem heart/body-weight ratio plotted as in C. Effect size interpretation (absolute value): no effect: (0), very small: (0.0,0.2), small: [0.2,0.5), medium: [0.5,0.8), large: [0.8,1.2), very large: [1.2,2.0), and huge: [2.0,∞)

Over the 15-month follow-up, both groups exhibited parallel, progressive weight gain (Fig. 2A), with statistical analyses yielding a non-significant effect size (95% CI encompassing zero), indicating no differences in weight trajectories.

CKD pigs exhibited an early and marked decline in glomerular filtration rate compared with controls (Fig. 2B), with statistically significant differences at 1.5 and 6 months (huge effect size). This impairment gradually diminished over time and was no longer evident at 15 months.

At study completion, the remaining left kidney displayed cortical thickening, an elevated arterial resistive index (Table S1; very large to huge effect size), and increased weight (huge effect size; Fig. 2C), consistent with compensatory hypertrophy. Figure 2Dshows an enlarged kidney with upper-pole surface scarring secondary to arterial branch clamping. In contrast, CKD animals showed reduced cardiac weight (Fig. 2E; huge effect size).

### CKD animals developed higher atherosclerosis burden

**Fig. 3:**
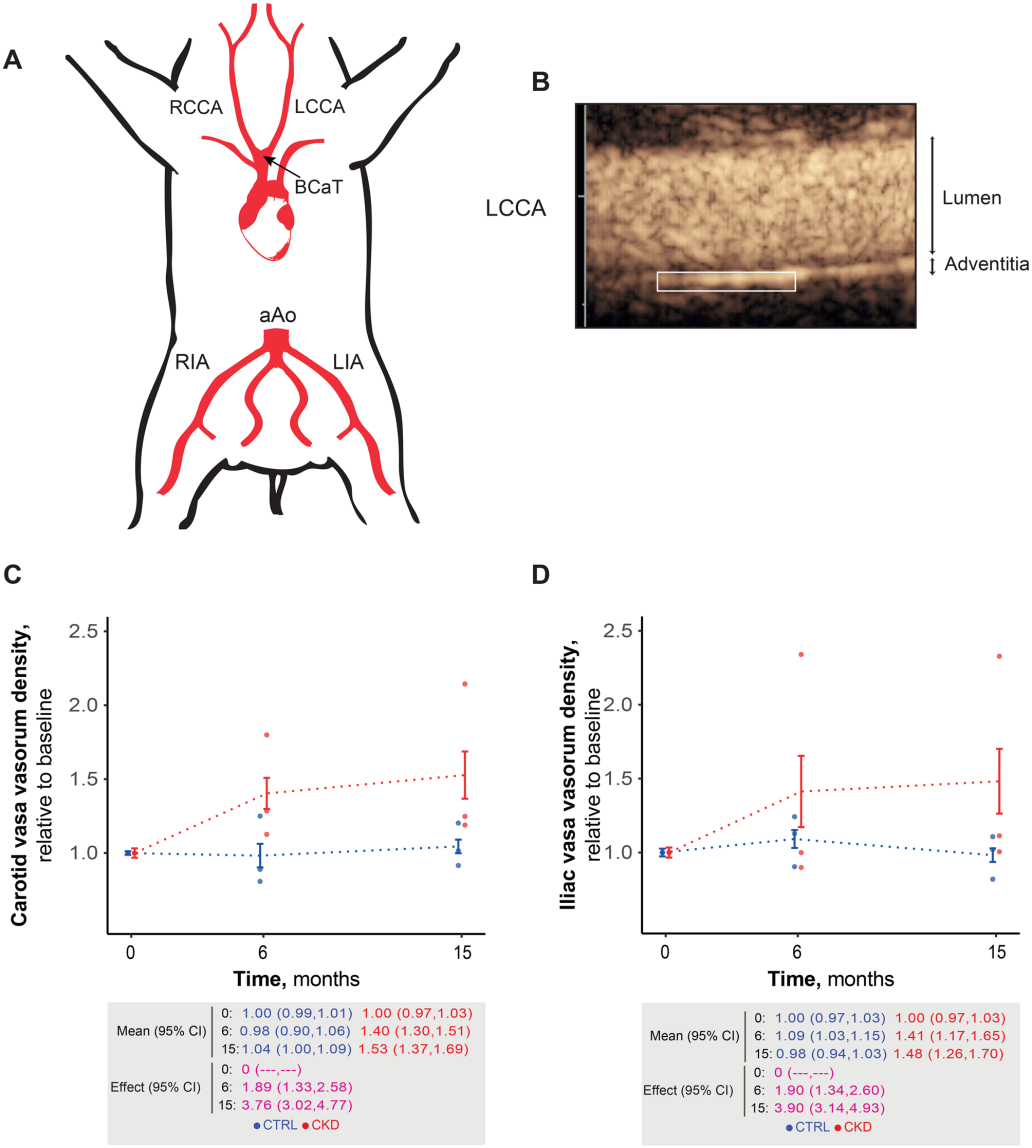
Adventitial vasa vasorum quantification in CKD pigs: (**A**): schematic of the porcine arterial tree, illustrating the ascending aorta (aAo), bicarotid artery trunk (BCaT), left and right common carotid arteries (LCCA, RCCA), and left and right iliac arteries (LIA, RIA). (**B**): representative contrast-enhanced ultrasound (CEUS) image of the LCCA, with adventitial vasa vasorum highlighted. (**C**): longitudinal assessment of carotid vasa vasorum, normalized to baseline over 15 months. Top: individual values, group means with 95% CI. Bottom: summary mean and effect size with 95% CI. (**D**): longitudinal assessment of iliac artery vasa vasorum, plotted as in C. Effect size interpretation (absolute value): no effect: (0), very small: (0.0,0.2), small: [0.2,0.5), medium: [0.5,0.8), large: [0.8,1.2), very large: [1.2,2.0), and huge: [2.0,∞)

Figure 3A provides an overview of the porcine arterial tree, highlighting the arterial territories assessed for atherosclerotic burden, including the carotid, coronary, abdominal aorta, and iliac arteries. A representative contrast-enhanced ultrasound (CEUS) image of the left common carotid artery with highlighted adventitial vasa vasorum is shown in Fig. 3B. Longitudinal in vivo CEUS demonstrated a progressive increase in adventitial vasa vasorum density in both the carotid and iliac arteries of CKD animals compared to controls (Fig. 3C–D), with effect sizes ranging from very large to huge.

**Fig. 4:**
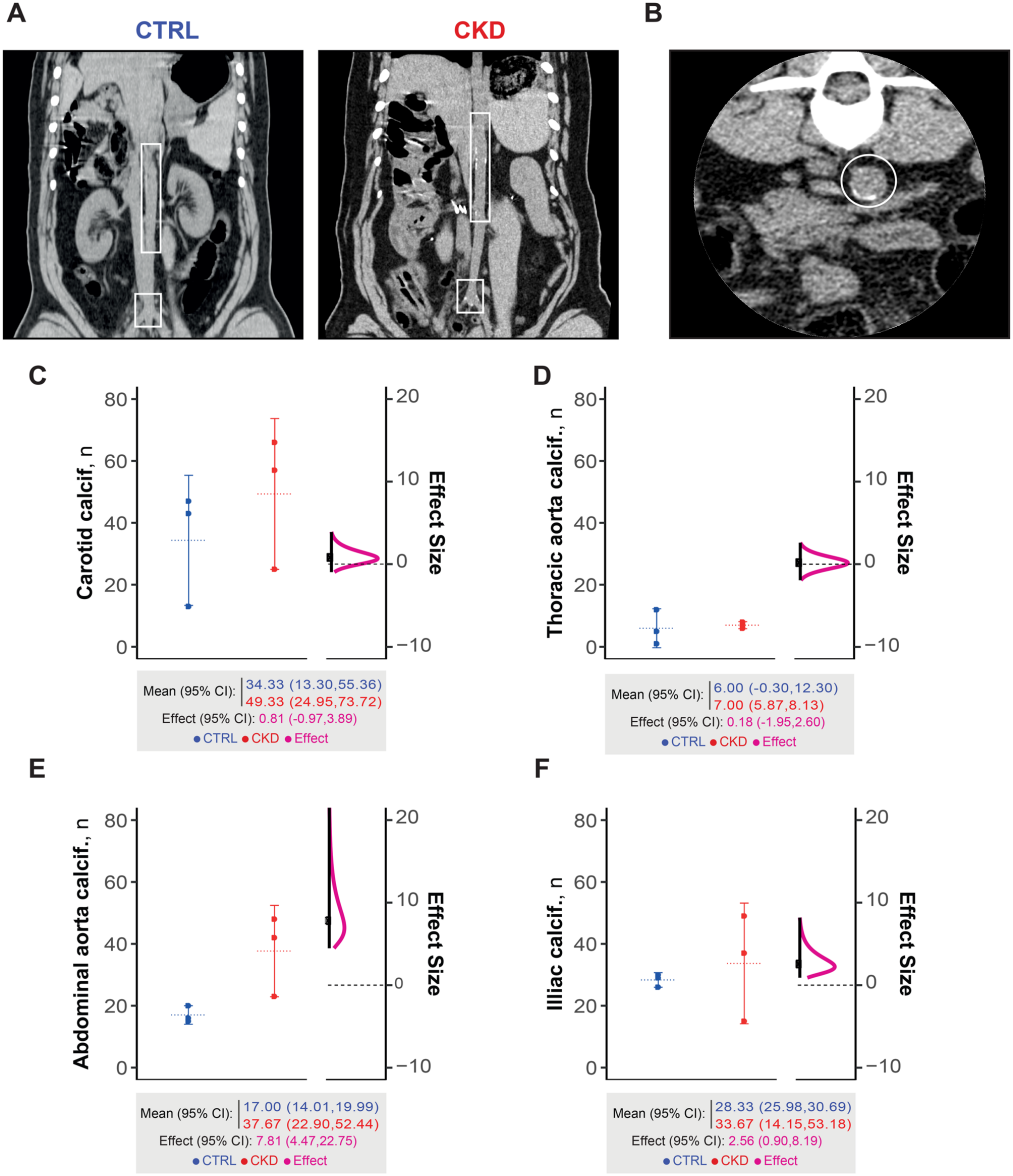
Visualization and quantification of vascular calcifications in CKD pigs: (**A**): contrast-free coronal CT reconstructions of the thoracoabdominal region. Vascular calcification foci are highlighted in the abdominal aorta (upper inset) and iliac arteries (lower inset). (**B**): axial CT slice of the abdominal aorta in CKD pigs, showing calcific deposits (circled). (**C**-**F**) quantification of arterial calcifications at 15 months in four vascular territories: common carotid artery (**C**), thoracic aorta (**D**), abdominal aorta (**E**), and iliac artery (**F**). For each panel, left plot displays individual values, group means with 95% CI. Right plots show distribution of effect size with 95% CI. Below each pair of plots, a summary of mean and effect size with 95% CI is provided. Effect size interpretation (absolute value): no effect: (0), very small: (0.0,0.2), small: [0.2,0.5), medium: [0.5,0.8), large: [0.8,1.2), very large: [1.2,2.0), and huge: [2.0,∞)

Contrast-free coronal CT reconstruction of the thoracoabdominal region at 15-month follow-up, illustrating vascular calcification foci, in CKD and control animals (Fig. 4A), with representative axial CT slice displaying abdominal aorta calcifications (Fig. 4B) are shown. Quantitative analysis confirmed a higher number of calcification foci in CKD animals within the abdominal aorta and iliac arteries (huge effect sizes; Fig. 4E–F); as well as an increased carotid artery blood flow velocity (huge effect size; Table S2), suggesting flow disturbances secondary to CKD-induced vascular remodeling.

**Fig. 5:**
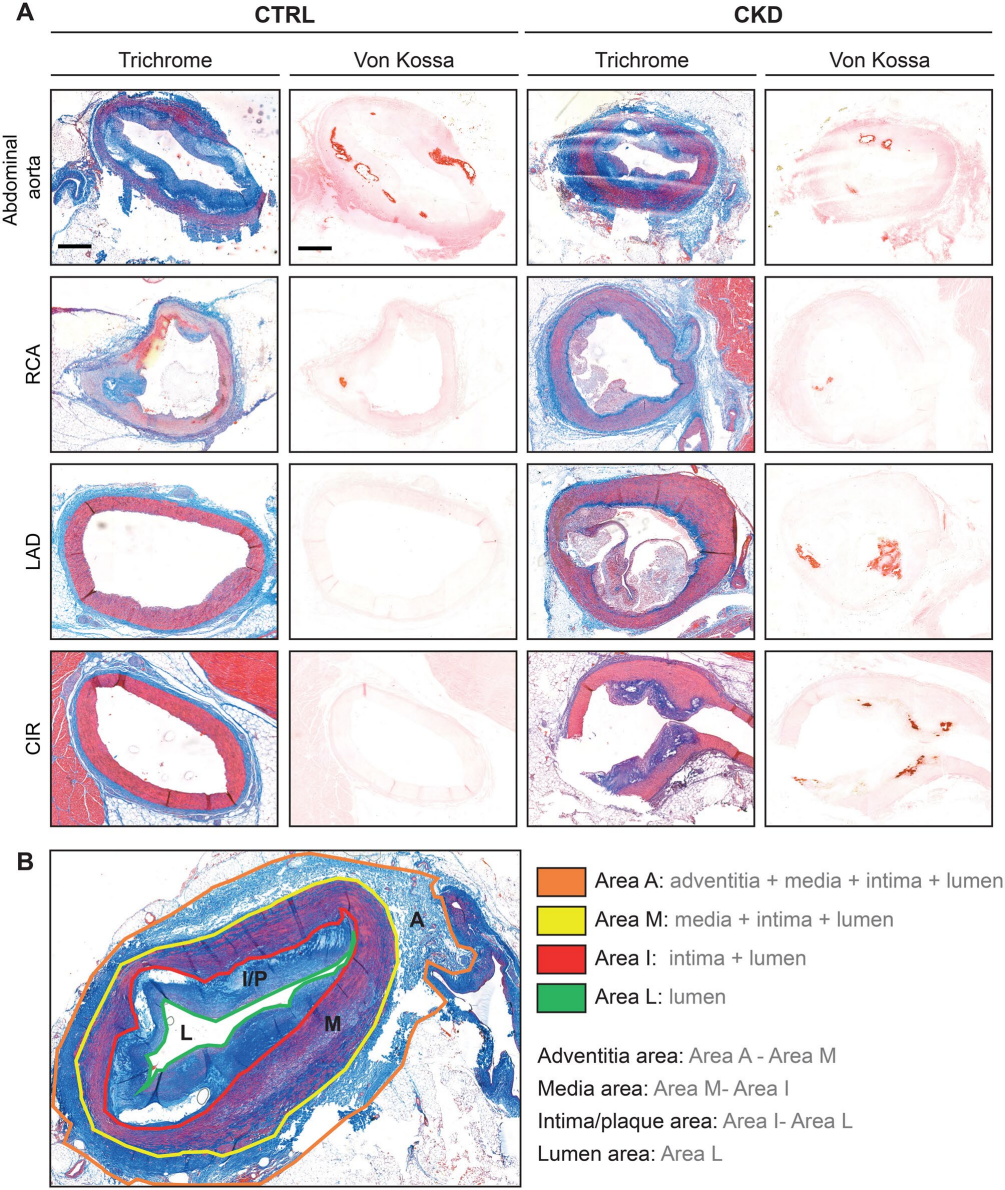
Atheromatous plaque characterization in CKD pigs: (**A**): Representative adjacent sections of the abdominal aorta and coronary arteries stained with Masson’s trichome (left) and Von Kossa (right). Scale bar = 2.5 mm. (**B**): schematic of morphometric areas used for quantification. Four concentric regions were delineated on trichome-stained sections: area A: between the border of the adventitia and surrounding tissue; area M: between the border of the media layer and the adventitia; area I: between the media layer and the intima layer, following the lamina media; and area L: between the intima layer and the lumen. The specific areas were calculated indirectly: intima/plaque area = area I - Area L; media layer area = area M - Area I; adventitia area = area a – area M

Post-mortem histological analyses revealed that coronary arteries had a greater atherosclerotic plaque and calcification areas in CKD animals (medium and huge effect sizes, respectively; Fig. 5 and Table 1). In addition, both the abdominal aorta and carotid arteries showed larger adventitial areas (very large and huge effect sizes; Fig. 5 and Table 1), consistent with the increased adventitial vasa vasorum density previously observed by CEUS in the carotid region.

**Table 1:** Artery histology

	CTRL	CKD	Effect size	
			Value	Interpretation
**Plaque area,** mm^2^				
Carotid artery	5.18 (2.25)	7.45 (4.03)	1.01 (−0.55,3.76)	*n.s*
Aorta	14.39 (8.49)	10.25 (4.69)	−0.49 (−1.49,0.39)	*n.s*
Coronary arteries	3.58 (3.76)	5.78 (6.08)	**0.59 (0.04,1.18)**	Medium
Circumflex	1.89 (2.42)	1.34 (1.85)	−0.23 (−1.30,0.78)	*n.s*
LAD	5.15 (5.08)	5.14 (5.71)	0.00 (−1.18,1.18)	*n.s*
RCA	3.95 (3.60)	10.29 (5.96)	**1.76 (0.87,3.07)**	Very large
**Media area,** mm^2^				
Carotid artery	29.54 (4.87)	33.09 (5.95)	0.73 (−0.90,3.23)	*n.s*
Aorta	18.27 (4.25)	22.76 (2.33)	**1.06 (0.19,2.18)**	Large
Coronary arteries	6.24 (9.67)	9.74 (11.32)	0.36 (−0.18,0.94)	*n.s*
Circumflex	2.04 (0.95)	2.17 (1.12)	0.14 (−0.88,1.19)	*n.s*
LAD	4.33 (3.28)	11.31 (9.23)	**2.13 (1.02,4.08)**	Huge
RCA	10.32 (13.57)	15.30 (14.45)	0.37 (−0.51,1.34)	*n.s*
**Adventitia area,** mm^2^				
Carotid artery	14.54 (4.61)	25.20 (15.75)	**2.31 (0.78,6.48)**	Huge
Aorta	12.38 (5.03)	18.60 (7.79)	**1.24 (0.37,2.41)**	Very large
Coronary arteries	7.96 (18.67)	3.67 (2.70)	−0.23 (−0.80,0.32)	*n.s*
Circumflex	1.79 (1.46)	1.58 (0.94)	−0.15 (−1.21,0.87)	*n.s*
LAD	2.90 (1.99)	3.75 (3.02)	0.43 (−0.68,1.71)	*n.s*
RCA	15.20 (27.02)	5.48 (2.28)	−0.36 (−1.33,0.52)	*n.s*
**Calcification area,** mm^2^				
Carotid artery	4494 (2799)	7053 (2469)	0.91 (−0.67,3.58)	*n.s*
Aorta	555850 (678919)	394302 (505275)	−0.24 (−1.36,0.80)	*n.s*
Coronary arteries	5169 (10417)	182533 (265646)	**17.03 (14.08,21.80)**	Huge
Circumflex	1865 (3565)	14732 (30835)	**3.61 (2.30,6.19)**	Huge
LAD	10150 (18005)	81185 (165458)	**3.95 (2.48,7.07)**	Huge
RCA	4113 (3453)	380609 (301558)	**109.02 (81.28,174.31)**	Huge

Values are shown as means and standard deviations. Effect size is shown as mean and 95% confidence interval. Significant effect sizes are shown in bold. Effect size interpretation (absolute value): no effect: (0), very small: (0.0,0.2), small: [0.2,0.5), medium: [0.5,0.8), large: [0.8,1.2), very large: [1.2,2.0), and huge: [2.0,∞). CKD: chronic kidney disease; CTRL: control. LAD: left anterior descending artery; n.s: no significant; RCA: right coronary artery

Overall, CKD animals developed a higher atherosclerotic burden than controls, characterized by (i) enhanced carotid vasa vasorum neovascularization, increased adventitial area, and altered blood flow, indicative of atherosclerotic lesion development; (ii) increased coronary plaque and calcification areas; (iii) greater calcification foci in the abdominal aorta and adventitial area; and (iv) augmented iliac arteries vasa vasorum neovascularization and calcification foci.

### Conventional blood and urine determinations

At study completion, CKD animals exhibited elevated blood creatine levels, reflecting impaired renal function (huge effect size; Table 2). In contrast, serum concentrations of calcium, phosphorous, sodium or potassium did not differ between CKD and control animals, suggesting preserved systematic electrolyte homeostasis.

**Table 2:** Conventional biochemical parameters

	CTRL	CKD	Effect size	
			Value	Interpretation
**Lipid profile**				
TG, mg/dL	45.00 (7.55)	65.67 (9.45)	**2.74 (1.04,8.65)**	Huge
TC, mg/dL	610.67 (63.58)	626.33 (161.99)	0.25 (−1.83,2.73)	*n.s*
HDL-C, mg/dL	142.33 (13.05)	136.33 (22.72)	−0.46 (−3.16,1.49)	*n.s*
LDL-C, mg/dL	459.33 (53.56)	476.87 (140.20)	0.33 (−1.70,2.89)	*n.s*
**Ions profile**				
Creatinine, mg/dL	1.74 (0.09)	2.34 (0.32)	**6.46 (3.62,18.92)**	Huge
Calcium, mg/dL	9.91 (0.29)	10.30 (0.19)	1.34 (−0.28,5.12)	*n.s*
Phosphorous, mg/dL	5.23 (0.56)	5.88 (0.77)	1.15 (−0.52,4.66)	*n.s*
Sodium, mmol/L	137.93 (1.92)	137.80 (1.39)	−0.07 (−2.39,2.14)	*n.s*
Potassium, mmol/L	3.98 (0.35)	3.99 (0.15)	0.04 (−2.20,2.34)	*n.s*
**Urine profile**				
Phosforous:Creatinine, mg/mg	0.116 (0.053)	0.327 (0.331)	**3.98 (1.97,12.01)**	Huge
Calcium:Creatinine, mg/mg	0.023 (0.021)	0.004 (0.001)	−0.90 (−4.11,0.83)	*n.s*
Choride:Creatinine, mmol/mg	0.246 (0.086)	0.230 (0.069)	−0.19 (−2.62,1.93)	*n.s*
Sodium:Creatinine, mmol/mg	0.232 (0.194)	0.220 (0.117)	−0.06 (−2.38,2.15)	*n.s*
Potassium:Creatinine, mmol/mg	0.321 (0.055)	0.274 (0.072)	−0.87 (−4.02,0.89)	*n.s*
Density, g/L	1.031 (0.006)	1.020 (0.017)	**-1.75 (−6.12,-0.16)**	Very large

Values are shown as means and standard deviations. Effect size is shown as mean and 95% confidence interval. Significant effect sizes are shown in bold. Effect size interpretation (absolute value): no effect: (0), very small: (0.0,0.2), small: [0.2,0.5), medium: [0.5,0.8), large: [0.8,1.2), very large: [1.2,2.0), and huge: [2.0,∞). C: cholesterol; CKD: chronic kidney disease; CTRL: control; HDL: high-density lipoprotein; LDL: low-density lipoprotein; n.s: no significant; TC: total cholesterol; TG: triglycerides

Conventional lipid profiling revealed a selective dyslipidemic pattern characterized by increased triglyceride levels (huge effect size; Table 2), while total cholesterol, high density lipoprotein cholesterol (HDL-C), and low-density lipoprotein (LDL-C) remained unchanged. Urine analysis further demonstrated a distinct renal functional phenotype, with increased phosphaturia and reduced urine density (huge and very large effect sizes; Table 2).

### CKD animals showed an altered inflammatory circulating profile

The circulating cytokine and chemokine profile is summarized in Table 3. CKD animals exhibited elevated serum concentrations of IL-1ra, IL-2, IL-4, IL-8, and IL-10 compared to controls (all showing huge effect sizes; Table 3), indicating a systemic immune response signalling.

**Table 3:** Cytokine and chemokine profile

	CTRL	CKD	Effect size	
			Value	Interpretation
**GM-CSF**, ng/mL	0.010 (0.000)	0.030 (0.035)	0.82 (−0.96,3.91)	*n.s*
**IFN-g**, ng/mL	1.92 (3.08)	0.45 (0.53)	−0.48 (−3.19,1.46)	*n.s*
**IL-1a**, ng/mL	0.010 (0.000)	0.047 (0.064)	0.82 (−0.96,3.91)	*n.s*
**IL-1b**, ng/mL	0.080 (0.000)	0.080 (0.000)	0.00 (–,–)	*n.s*
**IL-1ra**, ng/mL	0.047 (0.047)	0.220 (0.131)	**3.67 (1.75,11.15)**	Huge
**IL-2**, ng/mL	0.037 (0.038)	0.220 (0.145)	**4.84 (2.56,14.39)**	Huge
**IL-4**, ng/mL	0.067 (0.072)	0.880 (1.455)	**11.24 (6.59,32.52)**	Huge
**IL-6**, ng/mL	0.010 (0.000)	0.057 (0.057)	1.16 (−0.50,4.69)	*n.s*
**IL-8**, ng/mL	0.90 (1.25)	8.47 (12.68)	**6.06 (3.36,17.80)**	Huge
**IL-10**, ng/mL	0.030 (0.017)	0.200 (0.201)	**9.81 (5.71,28.45)**	Huge
**IL-12**, ng/mL	0.91 (0.51)	0.52 (0.09)	−0.77 (−3.81,1.02)	*n.s*
**IL-18**, ng/mL	0.203 (0.249)	0.313 (0.211)	0.44 (−1.51,3.12)	*n.s*
**TNF-a**, ng/mL	0.010 (0.000)	0.060 (0.070)	1.01 (−0.69,4.35)	*n.s*

Values are shown as means and standard deviations. Effect size is shown as mean and 95% confidence interval. Significant effect sizes are shown in bold. Effect size interpretation (absolute value): no effect: (0), very small: (0.0,0.2), small: [0.2,0.5), medium: [0.5,0.8), large: [0.8,1.2), very large: [1.2,2.0), and huge: [2.0,∞). CKD: chronic kidney disease; CTRL: control; GM-CSF: granulocyte macrophage-colony stimulating factor; IFN: interferon; IL: interleukin; n.s: no significant; TNF: Tumour necrosis factor; RA: receptor antagonist

### CKD animals developed a proatherogenic lipoprotein profile

The advanced lipoprotein profile is detailed in Table 4. In CKD animals, VLDL, LDL, and IDL particles exhibited increased triglyceride content (VLDL-TG, LDL-TG, and IDL-TG, respectively), while HDL particles showed elevated cholesterol content (HDL-C), all with huge effect sizes compared to controls.

**Table 4:** Advanced lipoprotein profile

			Effect size	
	CTRL	CKD	Value	Interpretation
**VLDL-P composition, mg/dL**				
VLDL-C	32.07 (6.41)	38.26 (7.32)	0.97 (−0.75,4.25)	*n.s*
VLDL-TG	38.16 (3.68)	52.93 (2.41)	**4.01 (1.99,12.09)**	Huge
Ratio VLDL-C/VLDL-TG	0.84 (0.15)	0.73 (0.16)	−0.76 (−3.80,1.03)	*n.s*
**VLDL-P number, nmol/L**				
Total	41.79 (5.01)	53.08 (3.42)	**2.25 (0.63,7.38)**	Huge
Large	1.097 (0.127)	1.404 (0.059)	**2.42 (0.78,7.83)**	Huge
Medium	5.16 (0.73)	6.51 (0.57)	**1.83 (0.24,6.32)**	Very large
Small	35.53 (4.21)	45.17 (2.83)	**2.29 (0.66,7.47)**	Huge
Ratio Large/Total	0.026 (0.001)	0.026 (0.001)	0.16 (−1.98,2.56)	*n.s*
Ratio Medium/Total	0.123 (0.006)	0.122 (0.004)	−0.15 (−2.55,2.00)	*n.s*
Ratio Small/Total	0.850 (0.005)	0.851 (0.004)	0.15 (−2.00,2.55)	*n.s*
Ratio VLDL-C/VLDL-P Total	0.76 (0.08)	0.72 (0.10)	−0.61 (−3.47,1.26)	*n.s*
**VLDL-P size, nm**	42.291 (0.017)	42.289 (0.022)	−0.14 (−2.53,2.01)	*n.s*
**LDL-P composition, mg/dL**				
LDL-C	408.13 (52.28)	402.68 (107.44)	−0.10 (−2.46,2.08)	*n.s*
LDL-TG	25.90 (2.28)	31.93 (8.69)	**2.65 (0.97,8.42)**	Huge
**LDL-P number, nmol/L**				
Total	3688.38 (454.76)	3735.85 (977.76)	0.10 (−2.08,2.46)	*n.s*
Large	588.67 (70.29)	582.11 (157.34)	−0.09 (−2.44,2.10)	*n.s*
Medium	1224.37 (158.30)	1208.97 (358.77)	−0.10 (−2.45,2.09)	*n.s*
Small	1875.34 (229.15)	1944.77 (465.62)	0.30 (−1.74,2.84)	*n.s*
Ratio Large/Total	0.160 (0.002)	0.156 (0.003)	**-1.71 (−6.00,-0.11)**	Very large
Ratio Medium/Total	0.332 (0.006)	0.321 (0.016)	**-1.63 (−5.81,-0.03)**	Very large
Ratio Small/Total	0.508 (0.004)	0.523 (0.017)	**3.60 (1.70,10.96)**	Huge
Ratio LDL-C/LDL-P Total	0.111 (0.001)	0.108 (0.001)	**-3.66 (−11.14,-1.75)**	Huge
**LDL-P size, nm**	21.41 (0.00)	21.35 (0.06)	**-20.69 (−59.58,-12.32)**	Huge
**IDL-P composition, mg/dL**				
IDL-C	22.57 (3.95)	23.43 (4.73)	0.22 (−1.88,2.68)	*n.s*
IDL-TG	8.52 (0.78)	10.93 (1.53)	**3.09 (1.32,9.60)**	Huge
**HDL-P composition, mg/dL**				
HDL-C	107.24 (0.79)	109.09 (21.65)	**2.34 (0.71,7.60)**	Huge
HDL-TG	9.19 (2.55)	12.44 (1.16)	1.27 (−0.37,4.96)	*n.s*
**HDL-P number, µmol/L**				
Total	41.91 (1.36)	43.57 (6.49)	1.22 (−0.43,4.83)	*n.s*
Large	0.494 (0.008)	0.500 (0.087)	0.85 (−0.92,3.98)	*n.s*
Medium	16.93 (0.51)	17.40 (2.48)	0.92 (−0.81,4.15)	*n.s*
Small	24.49 (1.49)	25.67 (3.94)	0.79 (−0.99,3.87)	*n.s*
Ratio Large/Total	0.012 (0.001)	0.011 (0.000)	−0.62 (−3.49,1.24)	*n.s*
Ratio Medium/Total	0.404 (0.018)	0.400 (0.005)	−0.26 (−2.75,1.82)	*n.s*
Ratio Small/Total	0.584 (0.019)	0.589 (0.005)	0.27 (−1.80,2.77)	*n.s*
Ratio HDL-C/HDL-P Total	2.56 (0.07)	2.49 (0.13)	−1.04 (−4.41,0.65)	*n.s*
**HDL-P size, nm**	8.373 (0.030)	8.364 (0.008)	−0.29 (−2.82,1.76)	*n.s*

Values are shown as means and standard deviations. Effect size is shown as mean and 95% confidence interval. Significant effect sizes are shown in bold. Effect size interpretation (absolute value): no effect: (0), very small: (0.0,0.2), small: [0.2,0.5), medium: [0.5,0.8), large: [0.8,1.2), very large: [1.2,2.0), and huge: [2.0,∞). C: cholesterol; CKD: chronic kidney disease; CTRL: control; HDL: high-density lipoprotein; IDL: intermediate-density lipoprotein; LDL: low-density lipoprotein; P: particle; n.s: no significant; TG: triglycerides; VLDL: very-low-density lipoprotein

Analysis of lipoprotein particle number revealed a marked increase in VLDL particle concentration (VLDL-P, very large to huge effect sizes) in CKD animal. In addition, average LDL particle size was reduced, driven by an increased proportion of small LDL particles (higher small/total LDL particle ratio) and a decreased proportion of medium and large LDL particles (lower medium/total and large/total LDL particle ratios, respectively, all very large to huge effect sizes).

### CKD animals had a specific lipidomic signature

**Fig. 6:**
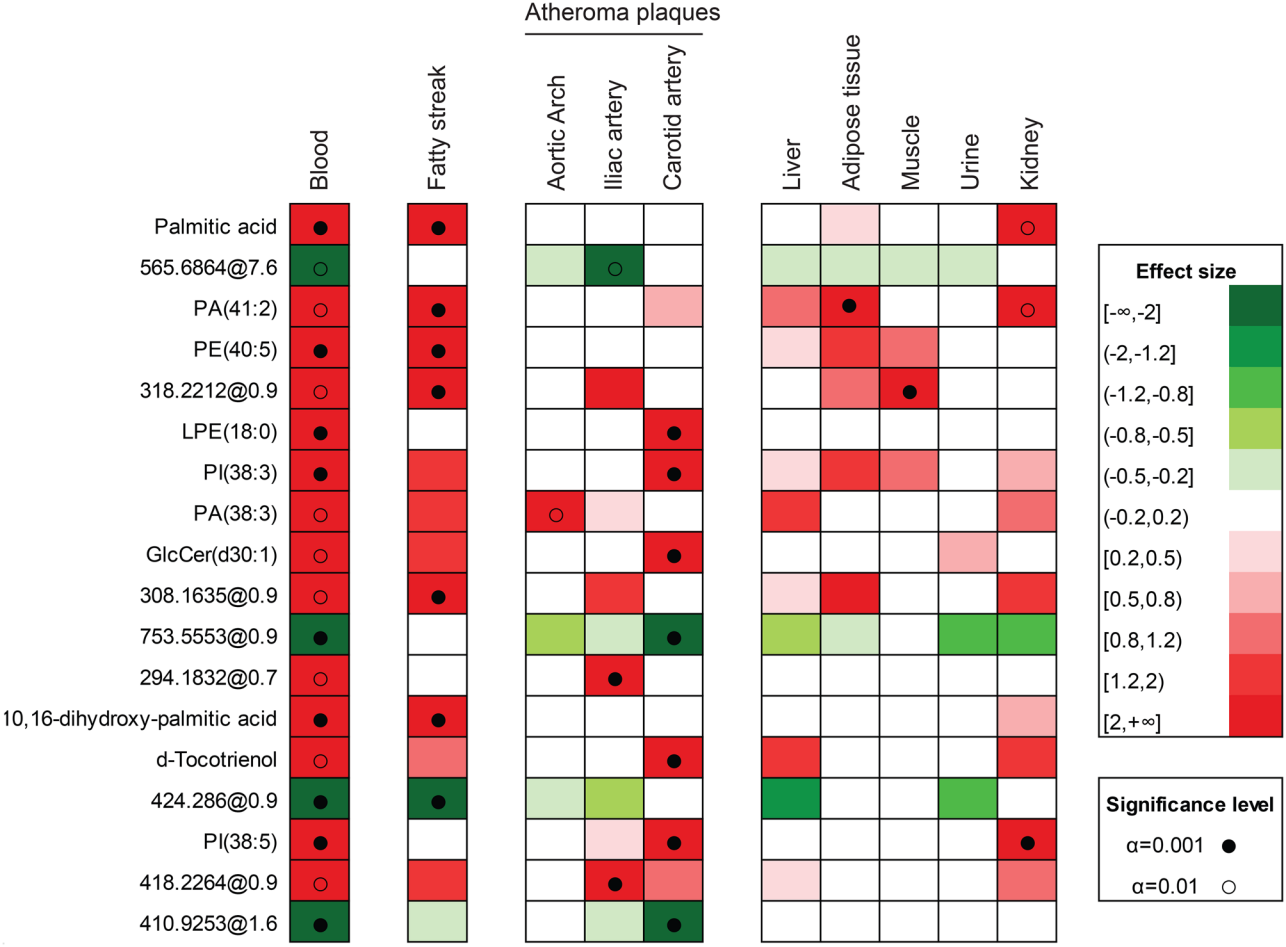
CKD-specific lipidomic signature: a total of 752 lipid species were quantified across serum and multiple tissues. Effect sizes with 99% CI confidence intervals were calculated to compare metabolite concentrations between control (CTRL) and CKD animals. Lipids exhibiting significant differences in serum and in at least one atheromatous lesion (fatty streak or atheroma plaque in aortic arch, iliac or carotid arteries), with consistent directionality, were selected. The abundance of these candidate metabolites was then explored in liver, adipose tissue, muscle, urine and kidney using the same criteria

The lipidomic fingerprint of CKD animals is shown in Fig. 6. Among 752 lipid species detected, 18 circulating compounds differed significantly between CKD and control animals at a 99% confidence level, both in blood and in at least one atheromatous lesion (fatty streak or atheroma plaque within the aortic arch, iliac artery or carotid artery). Of these, 10 species were accurately identified and 8 remained unclassified (Table S3).

In CKD animals, blood and kidney tissues exhibited increased levels of palmitic acid, phosphatidic acid (PA(41:2)), and phosphatidylinositol (PI(38:5)). Compositional alterations were also detected within atheromatous lesions. Fatty streaks accumulated palmitic acid, 10,16-dihydroxy-palmitic acid, phosphatidic acid (PA(41:2)), and phosphatidylethanolamine (PE(40:5); whereas advanced atheroma plaques were enriched in lysophosphatidylethanolamine (LPE(18:0)), phosphatidylinositol (PI(38:3) and PI(38:5)), phosphatidic acid (PA(38:3)), glucosylceramide (GlcCer(d30:1), and d-tocotrienol.

### CKD animals had a specific fatty acid composition shift

Figure S1 summarizes the alterations in fatty acid composition observed in CKD animals. The overall lipidome-derived fatty acid profile remained unchanged in systemic compartments, including liver, adipose tissue, muscle, urine, and serum level. In contrast, significant alterations were detected in atheromatous plaques, most prominently and uniquely within carotid artery lesions. These changes were characterised by an enrichment of polyunsaturated fatty acids from both the *n*-3 and *n*-6 series, as well as increased dimethyl acetal content. Notably, no fatty acid species displayed significant concordant directional changes (either increase or decrease) in both blood and arterial tissue.

## Discussion

Several surgical models of CKD in pigs have been previously established. Fist was the remnant kidney model developed by Misra et al., which combined staged renal artery embolization with contralateral partial nephrectomy in juvenile pigs and followed animals for 28–84 days [21]. Subsequently, Chade et al. described a bilateral renal artery stenosis model induced by endovascular coil placement, with 14 weeks of follow-up [22], which has been widely applied to investigate renal alterations and CKD-associated cardiac injury pigs [47–49]. A third model, developed by Liu et al. achieved 5/6 nephrectomy in Bama pigs through two consecutive laparoscopic procedures (partial followed by contralateral radical nephrectomy), identifying this approach as optimal for inducing CKD [23]. More recently, Mavropoulos et al. proposed a minimally invasive swine model of CKD-associated heart failure by staged renal artery embolization using autologous clot in Yorkshire pigs [24]. Despite these valuable contributions, previous swine CKD models primarily focused on renal pathophysiology or cardiorenal interactions. The direct impact of CKD on atherogenesis and plaque progression, has not been systematically investigated in those models.

In our model, CKD was induced by permanent, selective clamping of specific segmental branches of the left renal artery, immediately followed by total contralateral nephrectomy using a laparoscopic approach. This strategy deliberately preserves a proportion of functional parenchyma in the left kidney, thereby maintaining sufficient residual renal function after the nephrectomy. This model (with slight variations) has been extensively used to study CKD in rodents as it mimics the progressive renal failure after loss of renal mass in humans [50]. Glomerular filtration rate (GFR) was evaluated through plasma iohexol clearance [26], normalized to baseline values to quantify the surgery-induced decline. One and a half months post-intervention, the GFR had decreased by approximately 42%, but this reduction progressively attenuated over time (18% at 6 months and no significant reduction by study completion), reflecting compensatory hypertrophy on the remaining renal parenchyma. Similar adaptative responses have been reported in remnant kidney models, including increased kidney size and weight, augmented renal blood flow and volume [51]. Notably, despite apparent recovery of GFR to values comparable to baseline by the end of the study, plasma creatinine levels remained elevated, indicating persistent impairment of renal function [52]. In humans, CKD is defined as the presence of kidney damage or an estimated glomerular filtration rate below 60 mL/min/1.73 m^2^ regardless of cause [53]. Compared to the normal reference range for healthy young adults (100–120 mL/min/1.73 m^2^) this diagnostic threshold represents a 40–50% reduction in renal function, a decline similar to that observed in our CKD pig model.

In our CKD pigs, we observed a reduction in cardiac weight, which contrasts with previous reports describing cardiac hypertrophy as a hallmark of CKD in swine models. Earlier studies have shown that renal dysfunction is often accompanied by myocardial hypertrophy, fibrosis, and diastolic dysfunction [48]. Even mild CKD has been reported to induce alterations in mitochondrial and contractile proteins, increase oxidative stress, and promote extracellular matrix remodelling, leading to impaired cardiac perfusion and dysfunction [54]. Moreover, CKD is associated with coronary endothelial dysfunction caused by reduced oxide nitric (NO) bioavailability, which compromises myocardial perfusion at rest and during exercise [55]. Interestingly, the loss of NO bioavailability due to multiple comorbidities, such as diabetes mellitus, high-fat diet, or CKD, can be partially compensated by increased hydrogen peroxide-mediated vasodilation [56]. Additionally, CKD combined with left ventricular diastolic disfunction has been linked to altered expression of mitochondrial-related genes [49], and site-specific 5-methylcytosine changes in vascular endothelial growth factor (VEGF)-related genes, contributing to impaired angiogenesis and cardiac microvascular rarefaction [57]. Because we did not perform functional cardiac assessment in our animals, the unexpected reduction in heart weight cannot be fully interpreted. Further studies incorporating cardiac functional and structural analyses are warranted to elucidate the mechanisms underlying this finding and its clinical relevance in our CKD model.

The association between CKD and accelerated atherosclerosis has been well established in animal models. In genetically modified mice, such as ApoE and LDL receptor-deficient strains, as well as in rabbits, both with severe renal impairment (5/6 nephrectomy) and milder injury (uninephrectomy) have been shown to exacerbate atherogenesis [58–60]. In humans, CKD is recognized as an independent determinant of subclinical atherosclerosis, further supporting the casual link between renal dysfunction and vascular disease [2]. The transgenic Yucatan minipig expressing the D374Y gain-of-function mutant of human proprotein convertase subtilisin/kexin type 9 (PCSK9) represents a robust large-animal model of autosomal dominant hypercholesterolemia, characterized by marked hypercholesterolemia on a high-fat diet without cholic acid supplementation [10]. These animals develop advanced atherosclerotic lesions and plaque calcifications that closely resemble human disease, providing a relevant platform to study the interplay between CKD and atherogenesis.

Our CKD intervention group exhibited a progressive increase of adventitial vasa vasorum density in both carotid and iliac arteries. Several lines of evidence support microvascular dysfunction not only as an initiating factor but also as an amplifier mechanism of atherosclerotic progression [16]. Indeed, CKD animals displayed higher atherosclerotic burden, characterized by carotid flow disturbances and multiple calcification foci detected by computed tomography and confirmed by postmortem histopathological analysis. Vasa vasorum neoformation is closely linked to inflammatory processes and has been reported in diverse conditions, including HIV [61], chronic kidney disease [62], psoriasis [63], obesity [64], diabetes [65], non-alcoholic fatty liver disease [66], cardiovascular disease [67] and cardiovascular events [68]. In our model, CKD animals exhibited increased circulating levels of both pro-inflammatory (IL-2, IL-8) and anti-inflammatory (IL-1ra, IL-4, and IL-10) cytokines, compared with controls, indicating a complex inflammatory milieu that can promote fibrosis, tissue injury, and organ dysfunction [69, 70]. Previous, works described an increase in the expression of vascular endothelial growth factor-A (VEGF-A), macrophage migration inhibition factor (MIF), and matrix metalloproteinase-1 (MMP-1) in the porcine remnant kidney model [51].

CKD is strongly associated with dysregulated lipid metabolism, which contributes to disease progression [71]. In our CKD model, pigs exhibited hypertriglyceridemia similar to that observed in human CKD [72]. Advanced lipoprotein profiling unveiled profound alterations in particle number and composition, including increased triglyceride content in VLDL, LDL, and IDL particles, an accumulation of VLDL particles, and a reduction in LDL particle size. These findings are consistent with our previous observations in non-diabetic CKD patients [73], reinforcing the translational value of this model. Elevated levels of VLDLs, IDLs, and their remnants accelerate atherosclerosis progression by promoting cholesterol deposition within the arterial wall [74, 75]. Furthermore, small LDL particles can penetrate the endothelial barrier and undergo oxidative modification [76], further amplifying vascular injury and plaque development.

CKD animals showed a specific lipidomic signature, with significant alterations across multiple tissues, body fluids, and atherosclerotic lesions. Notable changes included serum triglyceride enrichment and remodeling of fatty acids (e.g., palmitic acid), glycerophospholipids (PA, PE, LPE, PI), sphingolipids (glucosylceramide), and prenol lipids (d-tocotrienol). Among these, glycerophospholipids, particularly PA, PI, and PE were the most affected, with potential consequences for membrane dynamics, lipid synthesis, cell signaling, differentiation, mitochondrial function, inflammation, fibrosis and apoptosis [77–83]. A key finding was the elevation of palmitate, which is known to induce endothelial dysfunction, endoplasmic reticulum stress, and autophagy inhibition [71, 84–89], all mechanisms implicated in atherosclerosis. Elevated circulating palmitate has been associated with a higher incidence of cardiovascular events [90], and greater dietary intake is linked to increased risk of coronary heart disease [91]. In our model, CKD animals showed increased palmitic acid and derivatives (e.g., 10,16-dihydroxy-palmitic acid) in blood and fatty streak regions, suggesting a potential role of palmitate in the initiation of atherosclerosis in CKD. These findings highlight the need to consider limiting dietary palmitic acid intake in CKD patients.

Our model offers several advantages that enhance its translational relevance for studying the interplay between CKD and atherosclerosis. A key advantage of our approach is the induction of stable CKD while preserving sufficient residual function to ensure long-term survival. In contrast, to embolization-based models or full 5/6 nephrectomy protocols, where extensive nephron loss aften leads to rapid uremia and limited viability, the selective, permanent clamping of segmental renal artery branches in our model produces a controlled and reproducible reduction of renal perfusion. This generates chronic hypoperfusion of defined cortical territories while maintaining enough functional parenchyma to allow extended follow-up, critical for evaluating chronic vascular processes. The physiological adaptations observed, including compensatory hypertrophy, mirror the clinical evolution of CKD more closely than models involving complete arterial occlusion or abrupt nephron loss [92]. The use of Yucatan minipigs and a single surgical episode also facilitates imaging feasibility, standardization and improved animal welfare. However, our approach requires advanced laparoscopic vascular expertise which may restrict adoption in centres without specialized surgical teams. Although our model induces CKD, it does not reach end-stage levels achievable in more aggressive surgical or embolization models, limiting its utility for studies focused on advanced uremia or dialysis-related physiology.

Our study has several limitations that warrant consideration. First, the small sample size may limit the statistical power and generalizability of our findings. Nonetheless, reporting effect sizes with 95% confidence intervals provides a transparent assessment of practical significance beyond traditional *p*-values [20]. Effect size is increasingly recognized as the most informative outcome of empirical studies, providing insight into the magnitude and clinical relevance of observed differences [38]. In this context, Weng and McV Messam emphasized the need to increase awareness in veterinary research regarding the reporting of statistical measures that quantify effect magnitude and associated uncertainty, advocating for their interpretation to guide clinical decision-making rather than focusing solely on *p*-values. Interestingly, they illustrated this approach using a hypothetical randomized controlled trial of dietary management in cats with CKD as a example [93]. However, we agree that future studies with larger cohorts and additional control groups will be necessary to validate and expand upon these findings. Second, blood pressure (BP) was not continuously monitored. Our facilities at the Institute of Biomedical Research of Lleida (CREBA) and the Centre for pig Studies lack BP telemetry, the gold standard for reliable and continuous BP assessment. Although non-invasive cuff-based methods are widely used for their convenience, they have shown poor agreement with direct arterial measurements in minipigs, affecting both systolic and diastolic readings [94]. Future studies should include validated BP measurements to better characterize hemodynamic status. Third, cardiac functional parameters were not evaluated. Consequently, the unexpected reduction in heart weight observed in CKD pigs cannot be fully interpreted without additional functional and histological data, which are essential for understanding myocardial remodelling. Finally, only female pigs were used in this study. Females were selected due to the technical difficulty of bladder catheterization in males, related to their long and oblique urethral course, as well as to reduce sex-related variability in body growth, physiology, and lipid metabolism. However, we acknowledge that this design may introduce a sex bias and could limit translational applicability. Sex differences are well documented in both CKD and atherosclerosis. Although CKD is more prevalent in women, men typically experience a faster decline in kidney function and are more likely to progress to kidney failure or die from cardiovascular disease, partly due to lifestyle factors and the detrimental renal effects of androgens [95]. Androgens have been shown to promote renal inflammation, fibrosis, activation of the renin-angiotensin-aldosterone system, oxidative stress and glomerular hypertension, all of which contribute to accelerated CKD progression in men [96]. In the context of atherosclerosis, men generally exhibit a greater plaque burden [97, 98], thinner fibrous caps, larger necrotic cores, and a pronounced inflammatory response in the arterial wall [99, 100]. Women, on the other hand, are relatively protected against atherosclerosis before menopause, likely due to hormonal and vascular factors, but tend to catch up with men after menopause, showing increased plaque vulnerability and cardiovascular risk [101]. These well-stablished sex differences underscore the importance of future studies incorporating male animals or comparing both sexes to enhance the translational relevance of our model.

In contrast, our study also presents several notable strengths. First, we developed a reproducible and minimally invasive large-animal model of CKD using selective clamping of renal artery branches combined with contralateral nephrectomy via a laparoscopic approach. This technique reduces perioperative morbidity compared to traditional open surgery while reducing renal functional impairment. Second, renal function was monitored using iohexol plasma clearance, a gold standard method for GFR assessment that is less biased than creatinine-based estimations and rarely used in preclinical pig models. Third, Yucatan minipigs allow the use of equipment designed for human use such as scanners and ultrasounds, which reinforces the translacionality of our model. Non-invasive imaging allowed the sequential characterization of CKD-induced atherosclerosis in animals. Fourth, the long-term follow-up (15 months) allowed us to characterize both acute and chronic adaptations of the remmant kidney, including compensatory hypertrophy and functional recovery, which are rarely explored in swine studies of CKD. Finally, the model reproduced key metabolic and morphological changes associated with CKD. Our pig CKD model mimics human CKD alterations, making it a translational model with high clinical potential.

In summary, our minimally invasive CKD pig model, characterized by reliable renal functional impairment and long-term follow-up provides a valuable platform for investigating renal adaptation and vascular consequences in CKD. Our current study demonstrated that CKD induces a distinct lipidomic signature and accelerates atherosclerosis progression in a novel minipig novel.

## Conclusions

Our minimaly invasive CKD surgical model in minipigs produced a pronounced decline in glomerular filtration, accompanied by increased circulating creatinine. CKD pigs exhibited progressive neovascularization of the adventitial vasa vasorum in both carotid and iliac arteries. CKD pigs developed an enhanced atherosclerotic burden, characterized by a greater number of calcification foci in the abdominal aorta and iliac arteries, as well as increased plaque and calcification areas in coronary arteries. CKD animals showed an altered inflammatory circulating profile. CKD pigs developed a distinct dyslipidemic phenotype including (i) elevated triglyceride levels; (ii) marked alterations in lipoprotein composition, particle number and size; (iii) a specific lipidomic signature in blood, tissues and atherosclerotic lesions; and (iv) a fatty acid composition shift in atherosclerotic plaques.

## Electronic supplementary material

Below is the link to the electronic supplementary material.

Supplementary material 1

## Data Availability

The data and materials supporting the findings of this study are available from the corresponding author upon reasonable request.

## Abbreviations

A: Adventitia
aAo: Ascending aorta
BCaT: Bicarotid artery trunk
C: Cholesterol
CaPB: Caudal polar branch
CEP: Centre for Pig Studies
CEUS: Contrast-enhanced ultrasound
CI: Confidence interval
CIR: Circumflex
CKD: Chronic kidney disease
CrPB: Cranial polar branch
CTRL: Control
GFR: Glomerular filtration rate
GlcCer: Glucosylceramide
GM-CSF: Granulocyte macrophage-colony stimulating factor
HDL: High-density lipoprotein
HF-HC: High-fat, high-cholesterol
I/P: Intima/plaque
IDL: Intermediate-density lipoprotein
IFN: Interferon
IL: Interleukin
L: Lumen
LAD: Left descending artery
LCCA: Left common carotid artery
LDL: Low-density lipoprotein
LIA: Left iliac artery
LPE: Lysophosphatidylethanolamine
LRA: Left renal artery
M: Media
n.s.: No significant
P: Particle
PA: Phosphatidic acid
PCSK9: Proprotein convertase subtilisin/kexin type 9
PE: Phosphatidylethanolamine
PI: Phosphatidylinositol
RA: Receptor antagonist
RCCA: Right common carotid artery
RIA: Right iliac artery
SD: Standard deviation
TC: Total cholesterol
TG: Triglycerides
TNF: Tumour necrosis factor
VLDL: Very-low-density lipoprotein
