# Supplementary material for: Chronic kidney disease induces a distinct lipidomic signature and accelerates atherosclerosis progression in a novel minipig model
Source: Lab Anim Res. 2026 Apr 2;42:10. doi: 10.1186/s42826-026-00274-w (PMC13045143; doi:10.1186/s42826-026-00274-w)
Supplement: Supplementary file 1 — Supplementary material 1 [file 42826_2026_274_MOESM1_ESM.docx]

**Additional material**

1. **Additional results**

**Table S1. Left kidney ultrasound examination**

|  | **CTRL** | **CKD** | **Effect size** | |
| --- | --- | --- | --- | --- |
|  |  |  | **Value** | **Interpretation** |
| **Cortical thickness**, cm |  |  |  |  |
| Upper pole | 0.99 (0.15) | 1.26 (0.19) | **1.73 (0.13,6.05)** | Very large |
| Lateral border | 0.85 (0.07) | 1.46 (0.47) | **8.55 (4.93,24.83)** | Huge |
| Lower pole | 0.80 (0.12) | 1.23 (0.52) | **3.37 (1.53,10.35)** | Huge |
|  |  |  |  |  |
| **Arterial resistive index (RI)** |  |  |  |  |
| Renal artery | 0.55 (0.39) | 1.48 (0.58) | **2.38 (0.74,7.71)** | Huge |
| Upper pole interlobar artery | 0.85 (0.27) | 1.75 (0.72) | **3.41 (1.56,10.45)** | Huge |
| Lower pole interlobar artery | 0.39 (0.02) | 0.71 (0.27) | **20.85 (11.73,77.88)** | Huge |

Values are shown as means and standard deviations. Effect size is shown as mean and 95% confidence interval. Significant effect sizes are shown in bold. Effect size interpretation (absolute value): no effect: (0), very small: (0.0,0.2), small: [0.2,0.5), medium: [0.5,0.8), large: [0.8,1.2), very large: [1.2,2.0), and huge: [2.0,∞). CKD: chronic kidney disease; CTRL: control; n.s: no significant.

**Table S2. Carotid artery blood Flow velocities**

|  | **CTRL** | **CKD** | **Effect size** | |
| --- | --- | --- | --- | --- |
|  |  |  | **Value** | **Interpretation** |
| **Left carotid diastolic velocity,** m/s | 9.43 (0.40) | 14.10 (4.59) | **11.55 (6.78,33.39)** | Huge |
| **Left carotid systolic velocity,** m/s | 36.63 (1.45) | 44.60 (17.55) | **5.49 (2.99,16.21)** | Huge |
|  |  |  |  |  |
| **Right carotid diastolic velocity,** m/s | 8.47 (1.27) | 13.63 (4.92) | **4.07 (2.03,12.25)** | Huge |
| **Right carotid systolic velocity,** m/s | 40.40 (5.88) | 39.23 (19.31) | -0.20 (-2.64,1.92) | *n.s* |

Values are shown as means and standard deviations. Effect size is shown as mean and 95% confidence interval. Significant effect sizes are shown in bold. Effect size interpretation (absolute value): no effect: (0), very small: (0.0,0.2), small: [0.2,0.5), medium: [0.5,0.8), large: [0.8,1.2), very large: [1.2,2.0), and huge: [2.0,∞). CKD: chronic kidney disease; CTRL: control; n.s: no significant.

**Table S3. Lipidomic compounds identified**

| **Compound** | **Ionization** | **Precursor m/z** | **Neutral Mass** | **Retention Time** | **Putative Compound** | **IdLevel** | **Category** | **Main Class** | **Sub Class** |
| --- | --- | --- | --- | --- | --- | --- | --- | --- | --- |
| L_N_256.2401@3.476993 | N | 2552328 | 2562401 | 3.5 | Palmitic acid | (2) ID exact mass and some peaks | Fatty Acyls [FA] | Fatty Acids and Conjugates [FA01] | Straight chain fatty acids [FA0101] |
| L_N_565.6864@7.635008 | N | 5646791 | 5656864 | 7.6 | Unknown |  |  |  |  |
| L_N_770.565@8.109988 | N | 7695577 | 770565 | 8.1 | PA(41:2) | (4) ID exact mass | Glycerophospholipids [GP] | Glycerophosphates [GP10] | Diacylglycerophosphates [GP1001] |
| L_N_793.5544@7.461025 | N | 7925471 | 7935544 | 7.5 | PE(40:5) | (1) ID exact mass and spectra | Glycerophospholipids [GP] | Glycerophosphoethanolamines [GP02] | Diacylglycerophosphoethanolamines [GP0201] |
| L_N_318.2212@0.9129979 | N | 3172139 | 3182212 | 0.9 | Unknown |  |  |  |  |
| L_N_481.3151@2.963005 | N | 4803078 | 4813151 | 3 | LPE(18:0) | (4) ID exact mass | Glycerophospholipids [GP] | Glycerophosphoethanolamines [GP02] | Monoacylglycerophosphoethanolamines [GP0205] |
| L_N_888.5679@6.828003 | N | 8875606 | 8885679 | 6.8 | PI(38:3) | (1) ID exact mass and spectra | Glycerophospholipids [GP] | Glycerophosphoinositols [GP06] | Diacylglycerophosphoinositols [GP0601] |
| L_N_762.4947@8.953997 | N | 7614874 | 7624947 | 9 | PA(38:3) | (4) ID exact mass | Glycerophospholipids [GP] | Glycerophosphates [GP10] | Diacylglycerophosphates [GP1001] |
| L_N_625.4969@7.464991 | N | 6244896 | 6254969 | 7.5 | GlcCer(d30:1) | (2) ID exact mass and some peaks | Sphingolipids [SP] | Neutral glycosphingolipids [SP05] | Simple Glc series [SP0501] |
| L_N_308.1635@0.9149985 | N | 3071562 | 3081635 | 0.9 | Unknown |  |  |  |  |
| L_N_753.5553@0.9120017 | N | 752548 | 7535553 | 0.9 | Unknown |  |  |  |  |
| L_N_294.1832@0.707998 | N | 2931759 | 2941832 | 0.7 | Unknown |  |  |  |  |
| L_N_270.2176@0.9140012 | N | 2692103 | 2702176 | 0.9 | 10,16-dihydroxy-palmitic acid | (2) ID exact mass and some peaks | Fatty Acyls [FA] | Fatty Acids and Conjugates [FA01] | Hydroxy fatty acids [FA0105] |
| L_N_396.3013@4.205004 | N | 395294 | 3963013 | 4.2 | d-Tocotrienol | (4) ID exact mass | Prenol Lipids [PR] | Quinones and hydroquinones [PR02] | Vitamin E [PR0202] |
| L_N_424.286@0.9239991 | N | 4232787 | 424286 | 0.9 | Unknown |  |  |  |  |
| L_N_884.541@6.306016 | N | 8835337 | 884541 | 6.3 | PI(38:5) | (1) ID exact mass and spectra | Glycerophospholipids [GP] | Glycerophosphoinositols [GP06] | Diacylglycerophosphoinositols [GP0601] |
| L_N_418.2264@0.9149975 | N | 4172191 | 4182264 | 0.9 | Unknown |  |  |  |  |
| L_N_410.9253@1.570005 | N | 409918 | 4109253 | 1.6 | Unknown |  |  |  |  |

GlcCer: glucosylceramide; LPE: lysophosphatidylethanolamine; PA: phosphatidic acid; PE: phosphatidylethanolamine; PI: phosphatidylinositol.
